# Setting weights for fifteen CHNRI criteria at the global and regional level using public stakeholders: an Amazon Mechanical Turk study

**DOI:** 10.7189/jogh.09.010702

**Published:** 2019-06

**Authors:** Kerri Wazny, John Ravenscroft, Kit Yee Chan, Diego G Bassani, Niall Anderson, Igor Rudan

**Affiliations:** 1 Centre for Global Health Research, Usher Institute of Population Health Sciences and Informatics, University of Edinburgh, Edinburgh, UK; 2 Moray House School of Education, University of Edinburgh, Edinburgh, UK; 3 Department of Pediatrics, Hospital for Sick Children and University of Toronto, Toronto, Ontario, Canada; 4 Centre for Global Child Health and SickKids Research Institute, Hospital for Sick Children, Toronto, Ontario, Canada; 5 Centre for Population Health Sciences, Usher Institute of Population Health Sciences and Informatics, University of Edinburgh, Edinburgh, UK

## Abstract

**Introduction:**

Stakeholder involvement has been described as an indispensable part of health research priority setting. Yet, more than 75% of the exercises using the Child Health and Nutrition Research Initiative (CHNRI) methodology have omitted the step involving stakeholders in priority setting. Those that have used stakeholders have rarely used the public, possibly due to the difficulty of assembling and/or accessing a public stakeholder group. In order to strengthen future exercises using the CHNRI methodology, we have used a public stakeholder group to weight 15 CHNRI criteria, and have explored regional differences or being a health stakeholder is influential, and whether the criteria are collapsible.

**Methods:**

Using Amazon Mechanical Turk (AMT), an online crowdsourcing platform, we collected demographic information and conducted a Likert-scale format survey about the importance of the CHNRI criteria from 1051 stakeholders. The Kruskal-Wallis test, with Dunn’s test for posthoc comparisons, was used to examine regional differences and Wilcoxon rank-sum test was used to analyse differences between stakeholders with health training/background and stakeholders without a health background and by region. A Factor Analysis (FA) was conducted on the criteria to identify the main domains connecting them. Criteria means were converted to weights.

**Results:**

There were regional differences in thirteen of fifteen criteria according to the Kruskal-Wallis test and differences in responses from health stakeholders vs those who were not in eleven of fifteen criteria using the Wilcoxon rank-sum test. Three components were identified: improve and impact results; implementation and affordability; and, study design and dissemination. A formula is provided to convert means to weights for future studies.

**Conclusion:**

In future CHNRI studies, researchers will need to ensure adequate representation from stakeholders to undue bias of CHNRI results. These results should be used in combination with other stakeholder groups, including government, donors, policy makers, and bilateral agencies. Global and regional stakeholder groups scored CHNRI criteria differently; due to this, researchers should consider which group to use in their CHNRI exercises.

Involvement of stakeholders in research priority setting processes has been described as “an indispensable part of the process of research prioritisation” [1]. Despite this acknowledgement, Nuyens found that at the time, no process that could adequately involve stakeholders had been developed or implemented [2]. Reviews of research priority setting exercises have demonstrated that participation of a wide range of stakeholders is necessary as stakeholders differ in their prioritisation of research options [1,3,4]. Indeed, a number of research prioritisation methods have aimed to include wider stakeholder groups in research prioritisation, including the Child Health and Nutrition Research Initiative (CHNRI), and the James Lind Alliance [4]. A comparison was conducted between priorities identified by the James Lind Alliance, which incorporates patients into priority-setting, and research being conducted. The authors found measurable differences, with research not informed by patients being much more likely to be drug-focused.

Although the categorisation of ‘stakeholder’ has been inconsistently applied by researchers, stakeholder groups listed as ‘important’ include researchers, methodologists, funding agencies, industry, patients/service users, policy makers, civil society, and members of the public [1,3,5,6]. Involvement of the public as a stakeholder group has been rare and difficult [3,5]. Barriers cited in systematic reviews exploring public and patient engagement in research included difficulty recruiting, public understanding of research methods, representation and representativeness, language and jargon, and logistics for the public and researchers (such as transportation and attendance, time constraints for the researchers and patients, and extra funding required to enable patient engagement) [7-9].

The CHNRI method was created in 2007 as a systematic and transparent research priority setting method that aims to be democratic through its inclusion of diverse stakeholder groups in addition to expert researchers. CHNRI has quickly become one of the dominant methods for research priority setting in global health [10]. It leverages knowledge of researchers to generate and score research priorities against a pre-set list of criteria. In the initial design of the CHNRI method, these criteria were intended to be weighted by a wider group of stakeholders to enable greater involvement from the wider health community and public [11].

In a recent review of the first 50 CHNRI exercises, over 75% did not involve stakeholders [5]. Almost half of those that did used weights set by Kapiriri et al. [6]. Kapiriri et al. conducted three exercises to weight the original five CHNRI criteria: (i) researchers, policy makers, and health practitioners through a Network were asked to rank the criteria on a scale of 1-5; (ii) a national-level exercise was conducted in South Africa asking academics, researchers, child/youth workers, teachers, a statistician, and members of the public to spend $100 USD across the five criteria; and, (iii) a questionnaire was administered to 20 participants at an international conference on child health [6]. Five of seven other exercises that utilised stakeholders were national-level exercises, which is a higher than expected proportion, given that 80% of CHNRI exercises have been conducted on a global level [5,10]. In addition to many past CHNRI exercises not using stakeholders, those that did were constrained by having 20 to 70 representatives and cannot be representative of wider stakeholder values [5]. This is especially the case in those exercises that did not include public participation, as the participant stakeholders represented occupations that are rare in society and therefore non-representative. Yoshida et al. believe that a possible reason for the low uptake of involving stakeholders in CHNRI exercises is that researchers would rather skip this step than use weights that are not representative of wider stakeholders’ views [5].The authors appeal for more stakeholder exercises to involve a larger number of stakeholders and, in particular, an exercise that can be representative of wider societal values [5].

As previous exercises to involve stakeholders have been limited by the number of non-expert participants and in the number of stakeholders involved [5,6,12-15], we have leveraged the Amazon Mechanical Turk (AMT), which is a crowdsourcing platform, to enable laypersons globally to rate the importance of CHNRI criteria. Because one of the benefits of the CHNRI method was the ability for research groups to tailor criteria to their exercises, we have chosen criteria that were used across more than one exercise and thus applicable to a range of conditions and more likely to be used in the future. Through AMT, we are re-weighting the CHNRI criteria using a crowd of predominantly laypersons who come from diverse backgrounds, representing different geographies, religions, and ethnicities. The results of this exercise can be leveraged to be more representative of the public than previous attempts and can be combined with weights from other stakeholders (ie, donors, policy makers, industry, etc.) which are easier to access to create stakeholder weights for upcoming exercises. Furthermore, we are exploring regional differences for each CHNRI criterion and whether, from the perspective of the public, the criteria appear too similar and can be collapsed into a smaller number of criteria.

## METHODS

### Data collection

We used a crowdsourcing platform, the Amazon Mechanical Turk (AMT), to collect information on demographics of a crowd and on their opinions on the importance of the CHNRI criteria. AMT employs workers, called ‘Turkers’, to perform micro-tasks or fill in surveys in exchange for small payments. Through AMT, researchers do not have access to the ‘crowd,’ but instead post their project (either a compilation of micro-tasks, such as image annotation, or a survey) and the payment offered, and AMT facilitates the exchange. AMT is beneficial for research as it is very rapid, does not facilitate a transfer of identifiable information (unless directly asked in a survey), and is inexpensive.

CHNRI criteria from the past 50 CHNRI exercises and one recent exercise [10,16] were used. While the original five CHNRI criteria remained unchanged as they are the most frequently used, additional criteria were combined or absorbed by the original five criteria when there was overlap. The CHNRI criteria were then transformed so that they could to be expressed as a question in easily understandable terminology. This was important because we expected at least some of the Turkers (AMT workers) to have English as a second language. A list of each CHNRI criteria and corresponding question can be found in **Table 1**.

**Table 1:** CHNRI Criteria and corresponding survey questions

Criterion	Question
Equity	How important is it for the research to help health access become more fair between people?
Disease burden reduction	How important is it for the research to result in less disease? For example, if researchers were studying heart disease, could they reduce people having heart attacks?
Answerability	How important is it for the researchers to be able to create a study to properly answer their research question?
Effectiveness	How important is it that the results of the research have an impact and will people (including doctors, nurses, and patients) actually use them?
Deliverability	How important is it that the results of the research are affordable to those who need them and to those who pay for the results (for example, the national or local government, or patients)?
Feasibility	How important is it for the researchers to have enough time, funding, and skilled staff to carry out the research?
Likelihood to fill a knowledge gap	How important is it for this research to result in new information?
Cost	How important is it for the results of this research to be less expensive than similar alternatives currently available? For example, if the research is looking at a drug for blood pressure, will the new drug be less expensive than the ones available now?
Sustainability	How important is it for the results to be long-lasting?
Acceptability/Issues surrounding use	How important is it for the research and the results of the research to be respectful to local beliefs and cultural practices?
Scale	How important is it that the results of the research will be widely available (for example, the results will be available throughout the country)?
Likelihood to attract national policy attention/Translational value	How important is it that the results of this research eventually turn into policy? For example, if a research is looking into a better way to identify diabetes, the government adopts the results and uses them to find people who have diabetes.
Implementation	How important is it that the intervention or results of this research can be changed to fit different groups of people (for example, different countries, regions in countries, or religions)? For example, medications that have cow-based products cannot be used in Hindu populations because of religious regions – is it important for medicines not to use cow-based products?
Technical possibility	How important is it that if the research involves technology, that the technology is easy and not expensive to develop?
Innovation	How important is it that the research is trying to make something better than what is currently being used?

Prior to completing the survey, Turkers were informed that this survey was for research purposes, the perceived risks and benefits of the survey, and that their participation should be voluntary. They were provided with the contact details of the lead author, if they had further questions about the survey. Turkers were instructed to stop the survey at any time prior to completion, should they wish to cease participation. Once their results were submitted, due to the anonymous nature of AMT and crowdsourcing, withdrawing data was not possible. Turkers were paid $1.75 USD for completing the survey.

We collected non-identifiable demographic data from each Turker. Surveys were distributed in batches at different times of the day to enable Turkers from different time-zones, and usied location-blocking to facilitate a more international response. In particular, users from the United States and India were asked not to participate in later stages of the study to ensure better global representation.

### Questions

A pilot survey was initially distributed to 20 Turkers, of which 19 were located in the United States and 1 in India. This survey included additional questions asking whether there were any difficulties understanding questions. The Turkers who answered the pilot test were paid $2.00 USD as they answered additional questions. As a result of the pilot test, one of the questions used to identify malicious workers was moved, and a question asking whether Turkers were health stakeholders was added.

The full survey can be found in the Interactive Online Tool in **Online Supplementary Document**. There were 6 questions intended to identify malicious workers. Malicious workers are those who indiscriminately answer survey questions, without actually reading the questions, thus introducing ‘noise’ in the data. As AMT provides payment for responses, Turkers have an incentive to answer as many surveys as possible, and malicious workers may select random responses to appear they are answering surveys. Thus, we introduced quality control questions to identify these Turkers. If a worker failed two or more of these questions, their responses were rejected and their data was not included in the analysis. Examples of questions intended to identify malicious workers include “please select the fourth star.”

CHNRI questions were scored on a Likert scale, from “not important at all’ to “very important” (0-5).

### Data analysis

Descriptive statistics were calculated for demographic characteristics. Several new variables were created. A proxy measure for immigration status was created using country of residence and country of birth; if these variables were the same, immigration status was ‘No,’ and if they were different, immigration status was coded as ‘Yes.’ New variables were created to organise countries of residence and countries of birth into the following World Bank regions: Latin America and the Caribbean; North America; Europe and Central Asia; East Asia and the Pacific; South Asia; Middle East and North Africa; and, Sub-Saharan Africa.

Kruskal-Wallis test was conducted to compare the means scores for each criteria between respondents from each World Bank region. Post-hoc tests were conducted using Dunn’s Test with a Bonferroni correction.

Wilcoxon rank-sum test was used to identify differences between those who identified as health stakeholders and those who did not, using simulations based on the Monte Carlo method

Factor Analysis (FA) was conducted on the CHNRI criteria to explore whether the criteria were similar enough to be collapsed into factors. The Bartlett’s test for sphericity indicated that correlations between the criterion were sufficient for a FA (X^2^ = 3755, df = 105, *P* < 0.001), and the Kaiser-Meyer-Olkin (KMO) index showed that sampling was adequate (KMO = 0.89), indicating that the data was suitable for a FA. Through examination of the scree plot, histogram and residuals, and using Juliffe’s criterion of 0.7 for the eigenvalues, 3 components were chosen for the FA. The components were rotated using an orthogonal rotation using varimax

Finally, means are converted to weights using the following formula:


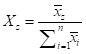


where X_z_ = the *z*^th^ weighted criterion, *x_i_* = the mean of the *i*^th^ criterion, and *n* is the total number of criteria in the exercise.

**Figure 1:**
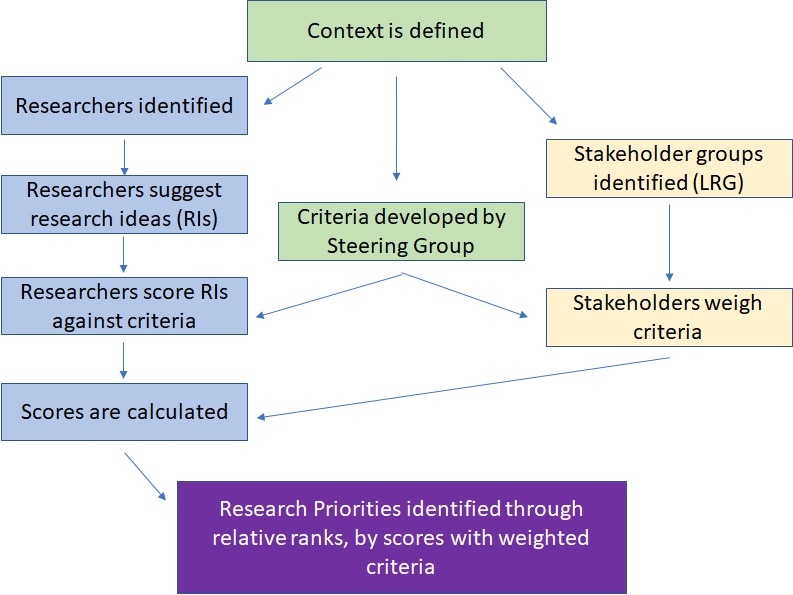
Flow diagram of a typical CHNRI exercise.

This formula is dependent upon the number of criteria a researcher chooses to use in his or her CHNRI exercise, and will be discussed further in the results section. **Figure 1** displays a flow diagram of a typical CHNRI exercise.

All analyses, except for calculating criteria weights, were computed in R Studio (R Studio, Boston, MA, USA) using R version 3.3.0.

There are two supplementary materials, both contained in **Online Supplementary Document**. One of them is Online Supplementary Material, containing tables, and the second is an Interactive Online Tool, containing an Excel form. The contents of both are described further in the results section.

## RESULTS

1051 individuals (pilot, n = 20, study, n = 1031) participated in the study, representing 73 countries and all 7 World Bank regions. The countries represented are displayed in Box S1 in the supplementary material in **Online Supplementary Document**. Demographic characteristics of the Turkers can be found in **Table 2****.**

**Table 2:** Demographic characteristics of respondents

Category	Number of participants (%)
**Residence in World Bank Regions:**	
Latin America & the Caribbean	133 (12.65)
North America	349 (33.21)
Europe & Central Asia	193 (18.36)
East Asia & the Pacific	70 (6.66)
South Asia	250 (23.79)
Middle East & North Africa	24 (2.28)
Sub-Saharan Africa	32 (3.04)
**Born in World Bank Regions:**	
Latin America & the Caribbean	143 (13.61)
North America	315 (29.97)
Europe & Central Asia	199 (18.94)
East Asia & the Pacific	68 (25.40)
South Asia	262 (24.93)
Middle East & North Africa	32 (3.04)
Sub-Saharan Africa	32 (3.04)
**Immigration status:**	
Immigrated – Yes	126 (11.99)
Immigrated – No	925 (88.01)
**Urban vs rural:**	
Urban	765 (72.79)
Rural	286 (27.21)
**Ethnicity:**	
Black African	51 (4.85)
Black Caribbean	10 (0.95)
Other Black	5 (0.48)
Central/South American	87 (8.28)
East Asian	36 (3.43)
South Asian	231 (21.98)
Southeast Asian	87 (8.28)
Middle Eastern	24 (2.28)
White	497 (4.29)
Multiple ethnicity	23 (2.19)
**Marital status**	
Married	416 (39.58)
In a domestic partnership or civil union	55 (5.23)
Single, but cohabiting with significant other	99 (9.42)
Separated	15 (1.43)
Divorced	21 (2.00)
Widowed	2 (0.19)
Single, never married	443 (42.15)
**Religion**	
Atheist/agnostic	271 (25.69)
Spiritual/non-religious	86 (8.18)
Buddhist	15 (1.43)
Catholic	182 (17.32)
Christian/Protestant/Methodist/ Lutheran/Baptist	172 (16.37)
Greek or Russian Orthodox	14 (1.33)
Hindu	188 (17.89)
Jewish	9 (0.86)
Mormon	2 (0.19)
Muslim	83 (7.90)
Other	29 (2.76)
**Employment:**	
Employed, working full-time	621 (59.09)
Employed, working part-time	128 (12.18)
Self-employed	121 (11.51)
Student	93 (8.85)
Not employed	73 (6.95)
Disabled, not able to work	5 (0.48)
Retired	10 (0.95)
**Health stakeholder:**	
Yes	269 (25.59)
No	762 (72.50)
Not available	20 (1.90)
**Education:**	
Primary school	2 (0.19)
Some high school	68 (6.47)
Some college, but no degree	151 (14.37)
College (2-year or technical college)	117 (11.13)
University (4-year degree)	423 (40.25)
Graduate-level degree (Masters or equivalent)	212 (20.17)
Professional degree (MD, J.D.)	53 (5.04)
Postgraduate degree (PhD)	25 (2.38)
**Political spectrum:**	
Extremely liberal	106 (10.09)
Moderately liberal	294 (27.97)
Slightly liberal	173 (16.46)
Neither liberal nor conservative	245 (23.31)
Slightly conservative	112 (10.66)
Moderately conservative	80 (7.61)
Extremely conservative	41 (3.90)
**Self-reported health status:**	
Excellent	194 (18.46)
Good	615 (58.52)
Neutral	188 (17.89)
Poor	46 (4.38)
Very poor	8 (0.76)
**Gender**:	
Male	689 (65.56)
Female	356 (33.87)
Non-binary	1 (0.10)
Other	5 (0.48)
**Age:**	
Minimum	18
Maximum	70
Inter-quartile range (IQR)	25.50 to 36.00
Mean	31.85
**Household size:**	
Minimum	1.0
Maximum	12.0
IQR	2.0 to 4.0
Mean	3.48

### Regional differences

There were significant differences, determined through the Kruskal-Wallis test, for all criteria except Answerability or Likelihood to fill a knowledge gap (**Table 3**).

**Table 3:** Means across criteria regionally and overall, Kruskal-Wallis test for significant differences

	Equity	Burden	Answerability	Effectiveness	Deliverability	Feasibility	Fill a Gap	Cost	Sustainability	Acceptability	Scale	Implementation	Translation	Technical	Innovation
Overall	4.37	4.42	4.40	4.36	4.32	4.41	4.09	3.75	4.05	3.11	4.29	3.17	3.62	3.84	4.33
Latin America & the Caribbean	4.47	4.39	4.44	4.53	4.29	4.52	4.20	3.82	4.08	3.10	4.34	2.91	3.69	3.99	4.48
North America	4.31	4.52	4.34	4.28	4.30	4.39	4.00	3.57	3.90	2.92	4.22	2.97	3.28	3.57	4.19
Europe & Central Asia	4.40	4.51	4.39	4.34	4.41	4.52	4.05	3.60	4.03	2.70	4.38	3.06	3.66	3.66	4.37
East Asia & the Pacific	4.51	4.57	4.54	4.53	4.46	4.60	4.30	4.17	4.34	3.18	4.50	3.10	3.84	4.13	4.50
Middle East & North Africa	4.58	4.13	4.33	4.08	4.38	4.33	3.88	3.67	3.79	3.58	4.33	3.38	3.96	4.04	4.54
Sub-Saharan Africa	4.66	4.72	4.69	4.75	4.63	4.81	4.16	4.28	4.38	3.00	4.72	3.44	3.72	4.38	4.66
South Asia	4.29	4.17	4.40	4.25	4.22	4.20	4.15	3.90	4.14	3.64	4.19	3.64	3.91	4.09	4.30
*P*-value*	0.004	<0.001	0.10	<0.001	0.02	<0.001	0.09	<0.001	0.001	<0.001	<0.001	<0.001	<0.001	<0.001	0.001

*P-values determined by the Kruskal-Wallis Test.

Significant differences among the equity criteria were driven by the comparisons between South America and East Asia and the Pacific (*P* = 0.03), and between Sub-Saharan Africa and South America (*P* = 0.04), with South Asians finding equity less important. Significant differences in disease burden reduction were driven by South Asia compared to East Asia and the Pacific (*P* = 0.002), Europe and Central Asia (*P* < 0.001), the North America (*P* < 0.001), and Sub-Saharan Africa (*P* < 0.001), with all South Asians finding disease burden reduction less important than the other regions. Comparisons between South Asia and other regions were also responsible for differences in the effectiveness criterion. Turkers residing in South Asia found effectiveness less important than those in Europe and Central Asia (*P* = 0.01), Latin America and the Caribbean (*P* = 0.001), and Sub-Saharan Africa (*P* < 0.001). In comparison to Latin America and the Caribbean, the Turkers from the Middle East and North Africa and North American regions found effectiveness less important (*P* = 0.03, *P* = 0.005, respectively). Additionally, Turkers from Sub-Saharan Africa found effectiveness significantly more important than those from the Middle East and North Africa, or from North America (*P* = 0.002, *P* = 0.002, respectively).

Turkers from South Asia found deliverability significantly less important than those from Europe and Central Asia (*P* = 0.05), and Sub-Saharan Africa (*P* = 0.04). South Asians also rated feasibility significantly less important than those from East Asia and the Pacific (*P* < 0.001), Europe and Central Asia (*P* < 0.001), Latin America and the Caribbean (*P* < 0.001), Sub-Saharan Africa (*P* < 0.001), and North America (*P* = 0.01). Moreover, in comparison to North Americans, Turkers residing in Sub-Saharan Africa rated feasibility significantly higher (*P* = 0.01).

Turkers residing in Europe or Central Asia ranked cost less important than those living in East Asia and Pacific (*P* < 0.001), Sub-Saharan Africa (*P* = 0.001)and those residing in South Asia (*P* = 0.04). North Americans rated cost significantly more important than those residing in East Asia and the Pacific (*P* < 0.001), South Asia (*P* = 0.01), and Sub-Saharan Africa (*P* < 0.001). Turkers living in East Asia and the Pacific rated sustainability higher than those in the Middle East and North Africa (*P* = 0.04), and North America (*P* = 0.01).

South Asian Turkers rated acceptability significantly higher than those in East Asia and the Pacific (*P* = 0.02), Europe and Central Asia (*P* < 0.001), Latin America and the Caribbean (*P* < 0.001), North Americans (*P* < 0.001), and South Asians (*P* = 0.02). Compared to Turkers from the Middle East and North Africa, those in Europe or Central Asia rated acceptability lower (*P* = 0.01).

Those in South Asia rated scale significantly less important than Turkers from East Asia and the Pacific (*P* = 0.04), Europe and Central Asia (*P* = 0.02), and Sub-Saharan Africa (*P* = 0.002). Turkers from Sub-Saharan Africa ranked scale more important than those in North America (*P* = 0.01). Implementation was ranked higher by South Asians than those residing in East Asia and the Pacific (*P* = 0.01), Europe and Central America (*P* < 0.001), Latin America and the Caribbean (*P* < 0.001), and North Americans (*P* < 0.001).

North Americans ranked translation into policy significantly lower than those residing in East Asia and the Pacific (*P* = 0.002), Europe and Central Asia (*P* = 0.004), Latin America and the Caribbean (*P* = 0.01), the Middle East and North Africa (*P* = 0.02), and South Asia (*P* < 0.001). North Americans also rated technical possibility less important than those residing in East Asia and the Pacific (*P* = 0.003), Latin America and the Caribbean (*P* = 0.003), South Asia (*P* < 0.001), and Sub-Saharan Africa (*P* < 0.001). South Asians ranked technical possibility significantly more important than those in Europe and Central Asia (*P* = 0.001). Finally, North Americans ranked innovation significantly less important than those residing in Latin America or the Caribbean (*P* = 0.03), or in Sub-Saharan Africa (*P* = 0.05).

### Stakeholders with health training/background

A previous CHNRI exercise found differences when comparing roles of health stakeholders [17]. Thus, we decided to explore whether being a stakeholder with health training/background would impact responses. The results from this comparison are displayed in the **Table 4**. When comparing results between those that identified as health stakeholders (ie, answered yes to working or studying in the health industry, the full question can be found in Box S2 in **Online Supplementary Document**), and those who did not, results were significantly different for all criteria except for cost, sustainability, technical possibility, and likelihood to fill a knowledge gap. Health stakeholders differed significantly from non-health stakeholders in their concern with criteria that related to the ability of research to reach its intended recipients and to be adequately carried out (ie, acceptability, implementation, technical possibility).

**Table 4:** Comparison of mean responses within each criterion from respondents who identified as being a health stakeholder and those who did not

	Equity	Burden	Answerability	Effectiveness	Deliverability	Feasibility	Fill a Gap	Cost	Sustainability	Acceptability	Scale	Implementation	Translation	Technical	Innovation
Health Stakeholder	4.29	4.20	4.34	4.21	4.22	4.23	4.12	3.83	4.09	3.62	4.18	3.55	3.83	3.97	4.26
Not Health Stakeholder	4.40	4.49	4.42	4.41	4.36	4.48	4.08	3.73	4.05	2.94	4.34	3.05	3.56	3.80	4.37
Mean Difference	-0.11	-0.29	-0.08	-0.20	-0.14	-0.25	0.04	0.10	0.04	0.68	-0.16	0.50	0.27	0.17	-0.11
Wilcoxon Rank-Sum Test	113780	123260	111870	118940	114220	121050	101150	99181	102790	71312	116330	80016	90930	95028	111440
*P*-value	<0.001	<0.001	0.0125	<0.001	0.002	<0.001	0.7334	0.4087	0.9382	<0.001	<0.001	<0.001	0.004	0.062	0.019

### Factor Analysis (FA)

Three components were identified through the FA, which together accounted for 49.37% of the total variance in the data. The results of the FA analysis are displayed in **Table 5** The first component (F1), which we summarise as improving and impacting results, contained criteria related to the importance of investing in health research that will result in genuine improvements to existing interventions, be effective, long-lasting, reduce the burden, fill a knowledge gap and be affordable in terms of absolute cost, end affordability, and cost of technological development and accounted for 18.39% of the variance. The second component (F2) accounted for 16.21% of the total variance and relates to implementation issues and affordability, and specifically relates to health research that is respectful to cultures and beliefs and can be altered to address the needs of diverse groups of people, will result in widespread policy adoption, and will be affordable in absolute cost and in technical design. Finally, the third component (F3), relates to study design and dissemination and accounted for 14.77% of the variance. F3 relates to ensuring studies are designed without methodological issues or practical issues (including funding, human resource or logistical issues), and that the research and results are equitable and the results are widely available and affordable.

**Table 5:** Results of factor analysis

CHNRI Criteria	Improve & impact results (F1)	Implementation & affordability (F2)	Study design & dissemination (F3)
Innovation	0.70		
Effectiveness	0.62		
Sustainability	0.60		
Burden	0.58		
Likelihood to fill a knowledge gap	0.56		
Acceptability		0.80	
Implementation		0.77	
Translation to policy		0.53	
Technical possibility	0.44	0.53	
Cost	0.47	0.51	
Equity			0.71
Answerability			0.66
Feasibility			0.65
Scale			0.59
Deliverability	0.42		0.47
Eigenvalues	2.76	2.43	2.22
% of variance	18.39	16.21	14.77

### Weighting

The mean weight for each criterion overall and by region are displayed in **Table 3****.** As the primary outcome for the manuscript is to develop relative weighting of the CHNRI criteria that can be used in future CHNRI exercises, the mean weights are not helpful in this format. However, the means can be used to calculate a relative weight. Because CHNRI exercises tend to differ in both the selection of criteria and the total number of criteria used, and weighting takes into account the number of criteria used and the relative means of the other criteria selected, it is not possible to present every possible scenario. In lieu of this, an example is provided and an interactive Excel (Microsoft Inc, Seattle WA, USA) sheet is available in **Online Supplementary Document**.

The formula to calculate the relative weights for each criterion is displayed in the methods section. The simply takes the mean score and divides by the sum of the mean scores for the criteria being used.

## DISCUSSION

This exercise had 1051 Turkers through AMT score the importance of fifteen CHNRI criteria using Likert scales. We provided a formula to convert the resulting means to weights, which can be used in future CHNRI exercises in order to involve public stakeholders.

There is an Excel sheet in the Interactive Online Tool in**Online Supplementary Document**. To use the interactive Excel sheet, navigate to the tab you wish to use (overall, or select a region). Place a ‘1’ in the “Choose?” column for all the criteria that you wish to use in your analysis. The weight column will automatically update with the weight for each criterion. Ensure that the ‘1s’ are placed in the “Choose?” column for *all* criteria that will be used in the exercise, as this affects the weights given for each criterion. These weights should not be used on their own to adjust the weights for the Research Priority Scores, as other stakeholder groups should be consulted, depending on your particular CHNRI exercise. CHNRI exercises should not solely use public stakeholder groups, but may also use stakeholder groups that contain physicians, patients, policy makers, donors, etc. The nature of the stakeholder groups will be context-dependent. Once the weights from other stakeholder groups are generated, a weighted average can be calculated for each criterion, then the weights applied to the scores within each criterion prior to calculating the weighted Research Priority Scores.

Despite highly left skewed data due to most criteria being ranked as ‘important’ or ‘very important’, regional differences in the distribution of responses for many criteria were detected when the data was analysed using Kruskal-Wallis test. Only two of fifteen criteria did not; these were answerability and likelihood to fill a gap in knowledge. In the thirteen criteria that showed regional differences, many of these differences appeared to be driven by South Asia and North America, which were the regions with the largest sample sizes (n = 250, and 349, respectively). Comparisons between other regions and Turkers from South Asia were responsible for the entirety of significant differences in four criteria (equity, disease burden reduction, deliverability, and implementation). Turkers in South Asia ranked equity, disease burden reduction, and deliverability significantly lower than other regions, while they ranked implementation significantly higher. Comparisons of Turkers from North America and other regions were wholly responsible for differences in the translation to policy and innovation criteria. North American Turkers ranked both these criteria lower than those from other regions. South Asians ranked implementation higher than 4 of the 6 other regions; this may be due to a targeted example in the definition, which asks about the importance to make non-bovine derived drugs to accommodate those who are Hindu (of which 70% of South Asian responders were, compared to 18% of total responders). Those from North America were primarily from the United States, where health policies in particular may have inequitable and unequal impact on the population, due to cost barriers, such as lack of insurance or high premiums. Those who are insured may have access to health care interventions that are not regional, state-wide, or national policies. This may explain the lower ranking of ‘translation to policy’ in this region compared to others.

Demonstrating regional differences underscores the importance of having regional representation from the global-level CHNRI exercises, for contributions both at the researcher-level and at the stakeholder-level, in order to ensure that voices are being heard. Moreover, if a national-level exercise is being conducted, it may be appropriate to ensure that only those from that nation are participating, as we have shown that there are differences in how the criteria are viewed and this could influence the results.

There were also differences between Turkers who answered ‘yes’ to whether they currently work or have worked in the health sector, and thus were coded as health stakeholders. While this question did not differentiate between specific types of health stakeholders and merely intended to identify if someone was a health stakeholder in the broadest sense possible compared to someone who was not, there were significant differences in eleven of fifteen criteria using the Wilcoxon rank-sum test. When looking at the mean differences (MDs), the largest MDs were in acceptability (0.68) and implementation (0.50), which are criteria that look at the importance of adapting research to other cultures and respecting other beliefs. Translating research into policy was also more important to health stakeholders than to non-health stakeholders (MD = -0.27). Health stakeholders were less likely to require research results to result in a reduction of disease (MD = -0.29) and found the effectiveness of the research results less important that non-health stakeholders (MD = -0.20). The latter is especially interesting, considering that research effectiveness relies heavily on the implementation and acceptability criteria; however, when examining the distributions, it appears that both health stakeholders and non-health stakeholders rated implementation and acceptability much lower than other criteria. As there are differences between results from those who identify as health stakeholders and those who do not, it underscores the importance of having stakeholder groups that can be representative of wider society. While it may be difficult to communicate CHNRI criteria to non-health stakeholders or explain the nature of a research cycle, the public’s values should be reflected in health research prioritisation in combination with other stakeholder groups.

We conducted a FA in order to explore the similarities between criteria and if any patterns would emerge from the data. We identified three components: improve and impact results (F1); implementation and affordability (F2); and, study design and dissemination (F3). These components show that, again despite having highly skewed data, there were clusters and patterns within responses that are interpretable. However, we would not recommend collapsing the criteria for expert scoring or Larger Reference Group (LRG) input, as we believe we would lose granularity that would be important when evaluating the research questions generated by CHNRI exercises. In the factor analysis, there was substantial cross-loading for technical possibility and cost, in factors 1 and 2. Factor 1, improving and impacting results, requires the research itself to be technically possible and cost-effective to conduct, as it would be impossible for the results of the research to be sustainable, effective, or to reduce disease burden, without satisfying these criteria. These two criteria were also found in Factor 2, which focused much more on the implementation of the research, and again requires the intervention to be possible given technology, and to have low costs to be optimally impactful.

In the past, it has been easier for CHNRI exercises to consult bilateral agencies, donors, government representatives and policy makers, for example, to conduct a weighting exercise, perhaps due to proximity to these stakeholder groups [5]. Our study is the first to set weights for a public, diverse, international stakeholder group on fifteen of the most commonly used CHNRI criteria and can provide a resource for future CHNRI studies. As our exercise used predominantly laypersons, the results should not be used to weight CHNRI criteria on their own, but should be combined with weights from other stakeholder groups including funders, bilateral agencies, members of government, policy makers, members of public with the disease or condition in question, family members of people with the disease or condition in question, and other relevant stakeholders.

### Limitations

While these studies were intended to be as global as possible and location-blocking features of AMT were used to attempt to minimise over-representation from certain countries, approximately one third of participants were from North America and one quarter from South Asia. Comparatively, only 2% of participants were from the Middle East and North Africa, 3% were from Sub-Saharan Africa, and 6% were from East Asia and the Pacific. Moreover, within regions, specific countries dominated responses. There were 73 respondents from Venezuela; the country within Latin America and the Caribbean with the next most frequent responses was Brazil with 17. India and the United States had 221 and 254 respondents, respectively. Still, we had at least one respondent from 73 countries.

Moreover, as with many crowdsourcing studies, there are concerns about the generalisability of the data. Since crowdsourcing surveys use ‘self-selected’ participants, their views may differ from those who would not opt to answer this type of survey. Moreover, Turkers must have access to technology as a prerequisite for participating in the study, as it is hosted online. This may make their experiences different from those without access to technology, or without knowledge of AMT, and the generalisability of the survey should be considered. Still, sampling views from over 1000 participants in over 70 countries in under two weeks is no small feat and would not be possible without access to crowdsourcing technology.

One concern with studies on online crowdsourcing platforms is verifying the validity of responses. While we employed questions to identify ‘malicious Turkers,’ which could identify whether a Turker was randomly selecting responses without reading the questions, we could not identify whether a Turker is deliberately answering incorrect answers. However, this would not be considerably different from other surveys collecting non-verifiable information.

Despite having over 1000 responses, we were unable to model interactions due to lack of power. Interactions, such as ethnicity and religion, or gender and ethnicity, could be important to consider.

While this study identifies the influence of demographic differences on Turkers’ views of the importance of each criterion, it is important to be mindful that the data are from a public stakeholder group. Other CHNRI exercises have shown that different stakeholder groups rate CHNRI criteria differently [6,15], and it may be possible that demographic differences within these stakeholder groups influence their views in different ways. It is important to conduct research in future on these different stakeholder groups to explore whether the patterns also exist, and will remain important for researchers conducting CHNRI exercise to report basic demographic information of stakeholder groups.

## CONCLUSION

As involvement of the public as a stakeholder group in health research priority setting has previously been difficult, we have provided a resource for future CHNRI exercises to use weights for fifteen CHNRI criteria globally and regionally. Because the relative importance of many criteria differed by region and whether respondents were health stakeholders, it will be important in future for researchers to choose their stakeholder samples carefully.

## Additional Material

Online Supplementary Document
