# Supplementary material for: Setting weights for fifteen CHNRI criteria at the global and regional level using public stakeholders: an Amazon Mechanical Turk study
Source: J Glob Health. 2019 Apr 2;9(1):010702. doi: 10.7189/jogh.09.010702 (PMC6445564; doi:10.7189/jogh.09.010702)
Supplement: Online Supplementary Document [file jogh-09-010702-s001.zip › Online Supplementary Material 1.docx]

***Online Supplementary Document***

| ***Supplementary Box S1****. Full survey, including instructions, demographic questions, CHNRI criteria and corresponding questions and questions intended to identify malicious Turkers.*  Thank you for participating in our survey on values for research prioritisation. We are collecting information on demographics (your age, where you live, etc.) and then on what you value most, so we will be able to apply your values to influence how funding agencies choose what to give money to when we set priorities for health research around the world. Your responses will be used to influence how money is spent on research in the future and could have great impact, so thank you! Participation is completely voluntary and we will not be able to identify you in any way, as is Amazon Mechanical Turk's policy. If you have any questions about the project, please contact kerri.wazny@ed.ac.uk.  **Individual and Demographic Questions**   \| Question \| Variable(s) \| \| --- \| --- \| \| Age \| What is your age? \| \| Gender \| What is your gender?  Options: Male, Female, Non-binary, Other \| \| Country of residence \| In what country do you live? \| \| Country of birth \| In what country were you born? \| \| Urban v. Rural \| Do you live in a large city or a town?  Options: Large city (more than 50,000 people live in your city); town (less than 50,000 people live in your town) \| \| Ethnicity \| What race/ethnicity best describes you? (Please choose only one)  Options: White, Black African, Black Caribbean, Other Black, Middle Eastern, Southeast Asian, East Asian, South Asian, Central/South American, Multiple Ethnicity \| \| Marital Status \| Which of the following best describes your marital status?  Options: Married, Widowed, Divorced, Separated, Common-law or domestic partnership/civil union, Single \| \| Religion \| What is your religion?  Options: Christian/Protestant/Methodist/Lutheran/Baptist, Catholic, Mormon, Greek or Russian Orthodox, Muslim, Hindu, Sikh, Buddhist, Judaism, Non-religious, Other \| \| Employment status \| What is your employment status?  Options: Self-employed, Employed part-time, Employed full-time, Student, Retired, Unemployed, Disabled (not able to work) \| \| Health stakeholder \| Do you work or have you worked in the health sector? (For example, as a doctor, nurse, in an organization that works in health through donations or research, as a health economist, dietician, sports therapist, etc.) \| \| Educational attainment \| What is your highest level of education?  Options: No formal education, Primary school, Some Secondary school, Secondary school, Some College, College (2 year degree), University (4 year degree), Graduate level degree (Masters or equivalent), Professional degree (i.e. J.D., M.D.), Postgraduate degree (PhD) \| \| Political affiliation \| When it comes to politics, would you describe yourself as liberal, conservative, or neither liberal nor conservative?  Options: Extremely liberal, moderately liberal, slightly liberal, neither liberal nor conservative, slightly conservative, moderately conservative, extremely conservative \| \| Overcrowding \| How many people currently live in your household?  (enter a number) \| \| Household earnings \| What is the total amount of *yearly* earnings for your *household*? (please enter a whole number in the currency of your country of residence) \| \| Self-reported health status \| How would you describe your health on a scale of 1-5, with 1 being very poor and 5 being excellent \|   **CHNRI Criteria:**  Instructions: Please use the scale of 1-5 to rank the following questions. The questions relate to research in health services, not just in your country, but throughout the world. Keep in mind that the values these questions refer to could include if you were participating in a study, what would be factors that would motivate you to participate or, generally, what kind of research you would think is important to do.   \| **Criterion** \| **Question** \| \| --- \| --- \| \| Equity \| How important is it for the research to help health access become more fair between people? \| \| Disease burden reduction \| How important is it for the research to result in less disease? For example, if researchers were studying heart disease, could they reduce people having heart attacks? \| \| Answerability \| How important is it for the researchers to be able to create a study to properly answer their research question? \| \| Effectiveness \| How important is it that the results of the research have an impact and will people (including doctors, nurses, and patients) actually use them? \| \| Deliverability \| How important is it that the results of the research are affordable to those who need them and to those who pay for the results (for example, the national or local government, or patients)? \| \| Feasibility \| How important is it for the researchers to have enough time, funding and skilled staff to carry out the research? \| \| Likelihood to fill a knowledge gap \| How important is it for this research to result in new information? \| \| Cost \| How important is it for the results of this research to be less expensive than similar alternatives currently available? For example, if the research is looking at a drug for blood pressure, will the new drug be less expensive than the ones available now? \| \| Sustainability \| How important is it for the results to be long-lasting? \| \| Acceptability/Issues surrounding use \| How important is it for the research and the results of the research to be respectful to local beliefs and cultural practices? \| \| Scale \| How important is it that the results of the research will be widely available (for example, the results will be available throughout the country)? \| \| Likelihood to attract national policy attention/Translational value \| How important is it that the results of this research eventually turn into policy? For example, if a research is looking into a better way to identify diabetes, the government adopts the results and uses them to find people who have diabetes. \| \| Implementation \| How important is it that the intervention or results of this research can be changed to fit different groups of people (for example, different countries, regions in countries, or religions)? For example, medications that have cow-based products cannot be used in Hindu populations because of religious reasons – is it important for medicines not to use cow-based products? \| \| Technical possibility \| How important is it that if the research involves technology, that the technology is easy to use and not expensive to develop? \| \| Innovation \| How important is it that the research is trying to make something better than what is currently being used? \|   **Malicious Worker Questions**  These questions will be scattered throughout the survey. The aim is to identify malicious workers and to exclude their answers from the analysis.   \| Please select number 3 \| Multiple choice or drop-down:  Options: 33, 4, 5, 3, 63 \| \| --- \| --- \| \| What is 1 + 1 \| Multiple choice or drop down:  Options: 1, 5, 11, 2, 8 \| \| What is this survey for? \| Multiple choice or drop down:  Options: animals, health, technology, environment \| \| Please select the star for ‘very important’ \| Star-scale (Likert scale) \| \| Please select the star for ‘slightly important’ \| Star-scale (Likert scale) \| \| Please select the fourth star \| Star-scale (Likert scale) \| |
| --- | --- | --- | --- | --- | --- | --- | --- | --- | --- | --- | --- | --- | --- | --- | --- | --- | --- | --- | --- | --- | --- | --- | --- | --- | --- | --- | --- | --- | --- | --- | --- | --- | --- | --- | --- | --- | --- | --- | --- | --- | --- | --- | --- | --- | --- | --- | --- | --- | --- | --- | --- | --- | --- | --- | --- | --- | --- | --- | --- | --- | --- | --- | --- | --- | --- | --- | --- | --- | --- | --- | --- | --- | --- | --- | --- | --- |
